# Molecular surveillance of anti-malarial resistance *Pfdhfr* and *Pfdhps* polymorphisms in African and Southeast Asia *Plasmodium falciparum* imported parasites to Wuhan, China

**DOI:** 10.1186/s12936-020-03509-w

**Published:** 2020-11-25

**Authors:** Tingting Jiang, Weijia Cheng, Yi Yao, Huabing Tan, Kai Wu, Jian Li

**Affiliations:** 1 Department of Human Parasitology, School of Basic Medical Sciences, Hubei University of Medicine, Shiyan, 442000 China; 2 Department of Infectious Diseases, Renmin Hospital, Hubei University of Medicine, Shiyan, 442000 China; 3 Department of Schistosomiasis and Endemic Diseases, Wuhan City Center for Disease Prevention and Control, Wuhan, 430015 China

**Keywords:** *Plasmodium falciparum*, Sulfadoxine/pyrimethamine, Antimalarial drug resistance, Dihydrofolate reductase, Dihydropteroate synthase, Mutation

## Abstract

**Background:**

Anti-malarial drug resistance is a severe challenge for eventual control and global elimination of malaria. Resistance to sulfadoxine-pyrimethamine (SP) increases as mutations accumulate in the *Pfdhfr* and *Pfdhps* genes. This study aimed to assess the polymorphisms and prevalence of mutation in these genes in the *Plasmodium falciparum* infecting migrant workers returning to Wuhan, China.

**Methods:**

Blood samples were collected for 9 years (2011–2019). Parasite genomic DNA was extracted from blood spots on filter paper. The mutations were evaluated by nested PCR and sequencing. The single-nucleotide polymorphisms (SNPs) and haplotypes of the *Pfdhfr* and *Pfdhps* genes were analysed.

**Results:**

*Pfdhfr* codon 108 showed a 94.7% mutation rate, while for *Pfdhps*, the rate for codon 437 was 79.0%. In total, five unique haplotypes at the *Pfdhfr* locus and 11 haplotypes at the *Pfdhps* locus were found while the *Pfdhfr*-*Pfdhps* combined loci revealed 28 unique haplotypes. A triple mutant (**IRN**I) of *Pfdhfr* was the most prevalent haplotype (84.4%). For *Pfdhps*, a single mutant (S**G**KAA) and a double mutant (S**GE**AA) were detected at frequencies of 37.8 and 22.3%, respectively. Among the combined haplotypes, a quadruple mutant **(IRN**I-S**G**KAA) was the most common, with a 30.0% frequency, followed by a quintuplet mutant (**IRN**I-S**GE**AA) with a frequency of 20.4%.

**Conclusion:**

The high prevalence and saturation of *Pfdhfr* haplotypes and the medium prevalence of *Pfdhps* haplotypes demonstrated in the present data will provide support for predicting the status and progression of antifolate resistance in malaria-endemic regions and imported malaria in nonendemic areas. Additional interventions to evaluate and prevent SP resistance should be continuously considered.

## Background

Malaria is caused by the *Plasmodium* parasite, which is transmitted to human beings via the bites of infected female *Anopheles* mosquitoes. It is prevalent in the tropics and subtropics, particularly sub-Saharan Africa, as well as in Southeast Asia (SEA) and South America. In 2018, there were an estimated 228 million new cases of malaria, which was responsible for approximately 405,000 deaths [1]. Among them, pregnant women and children under 5 years old in Africa are thought to be the primary victims.

In the 1980s, sulfadoxine-pyrimethamine (SP) replaced chloroquine (CQ) as the front-line anti-malaria treatment when large-scale CQ resistance developed in sub-Saharan African countries. However, SP soon had to be replaced by artemisinin-based combination therapy (ACT) due to drug resistance. However, SP is still used for intermittent preventive treatment in infants (IPTi) and pregnant women (IPTp) during malaria-endemic regions, following the guidance of the World Health Organization (WHO) [2]. Furthermore, the administration of SP plus amodiaquine is applied for seasonal malaria chemoprevention (SMC) [3]. Currently, the emergence, development, and continuous dissemination of *Plasmodium falciparum* resistance to the anti-malarial drug is considered a significant global threat for malaria control and elimination strategies [4]. The development of drug resistance could be influenced by multiple factors, including mutation frequency, treatment costs, drug selection pressure, patient compliance, and host immunity [5, 6]. It is necessary to conduct molecular epidemiological surveillance and monitoring of drug-resistant *P. falciparum* parasites from disease-endemic to nonendemic areas. Molecular markers are a useful tool for confirming that parasites are drug-resistant.

For *Plasmodium* spp*.*, enzymes involved in folate metabolism are interfered with by the antifolate anti-malarial drugs. Pyrimethamine acts as an inhibitor in *P. falciparum* dihydrofolate reductase (*Pfdhfr*) and sulfadoxine, targets the *P. falciparum* enzyme dihydropteroate synthase (*Pfdhps*) [7]. In vitro and in vivo studies have demonstrated resistance to SP is mainly mediated by mutations at codons *Pfdhfr* N51**I**, C59**R**, S108**N,** and I164**L,** and *Pfdhps* S436**A**, A437**G**, K540**E**, A581**G,** and A613**S** [8, 9]. SP resistance, with is very common, is accompanied by the accumulation of these mutations [10–12]. In particular, combinations of multiple mutations in both genes, such as the quadruple mutant carrying four partially resistant mutation in combination, are mainly comprised of the *Pfdhfr* triple mutant (N51**I**/C59**R**/S108**N**) and *Pfdhps* (A437**G**). The quintuple mutant genotype includes the *Pfdhfr* (N51**I**/C59**R**/S108**N**) and *Pfdhps* (A437**G**/K540**E**). The sextuple mutant consists of a triple mutant (N51**I**/C59**R**/S108**N**) in *Pfdhfr* and a triple mutant (A437**G**/ K540**E**/A581**G**) in *Pfdhps*, a combination that was called super resistant [13–15]. Multiple combinations of mutations can affect IPTi and IPTp treatment outcomes. Therefore, it is necessary not only to monitor the increase of mutations at a single site, but also to prevent the potential combination of more other multiple mutations.

This study investigated the prevalence of the mutant and wild-type alleles isolated from *P. falciparum* infecting migrant workers who have returned to Wuhan, central China, who all came from perennial transmission regions from 2011–2019. Such molecular surveillance will provide health authorities with valuable information for adopting efficient anti-malarial drugs in malaria-endemic regions in Africa and malaria nonendemic areas with imported malaria in China particularly Wuhan.

## Methods

### Samples collection

Blood samples were collected from *P. falciparum*-infected migrant patients with uncomplicated malaria in Wuhan of Hubei Province from 2011 to 2019. These samples were examined by microscopy with stained thick and thin blood smears and detected by rapid diagnostic tests (RDTs) for Pf-HRP2 and pLDH, as previously described [16–18]. RDTs, were carried out according to the manufacturer's manual (Wondfo, Guangzhou, China). Subsequently, two or three drops of blood were spotted on Whatman 3MM filter paper, air dried, and stored in an individually coded sealed plastic bag containing silica desiccant beads. The bags were stored at 4 °C until use. The study was approved by the Ethical Review Committee of the Hubei University of Medicine and Wuhan City Center for Disease Prevention and Control Ethics Committee. Informed consent was obtained and signed by all participants or their guardians before inclusion in the study and before sample collection.

### Molecular procedures

Genomic DNA (gDNA) from dried blood spot samples was isolated using the TIANamp Blood DNA Kit (Tiangen Biotech Co., Ltd., Beijing, China) following the manufacturer’s recommendations. DNA samples were stored at − 20 °C for further genotyping.

To identify polymorphisms in the *Pfdhfr* (Gene ID: PF3D7_0417200) and *Pfdhps* (Gene ID: PF3D7_0810880) genes, purified gDNA templates were amplified by using nested PCR in the Mini MJ Thermal Cycler (Bio-Rad), following protocols described previously with minor changes [19, 20]. Briefly, primary PCR, was performed in a total of 20 μl containing 10 μl of 2 × Phusion PCR Master Mix (40 units/ml Phusion DNA polymerase, 400 μM deoxynucleoside triphosphate [dNTP] mixture, 2 × Phusion high-fidelity [HF] buffer, and 3 mM Mg^2+^), 1 μl of each primer (10 μM), and 2 μl of gDNA. For the second round, 1.0 μl of primary PCR products were amplified with a 50 μl reaction system. All PCR conditions conducted were as follows: predenaturation at 95 °C for 3 min, followed by 30 cycles of denaturing for 30 s at 95 °C, annealing for 30 s at 55 °C and extension at 72 °C for 30 s, plus a final extension at 72 °C for 5 min. The PCR product was electrophoresed on a 1.0% agarose gel stained with GelRed®Safe DNA Gel Stain (Boitium, USA) and visualized with a UV transilluminator. Sequencing was outsourced to Genewiz, Soochow, China, whereby for the *Pfdhfr* gene, the nested PCR products were purified and sequencing in reverse directions, while for the *Pfdhps* gene, the nested PCR products were purified and sequenced by Bidirectional DNA sequencing. Polymorphisms were analysed by creating consensus nucleotide sequences with the reference sequences in PlasmoDB using DNAStar (DNASTAR Inc., Madison, WI, USA).

### Statistical analysis

The frequencies of single-nucleotide polymorphisms (SNPs) and haplotypes were calculated as the percentage of the number of successful sequencing samples. Mixed infections containing wild-type and mutation were excluded from further analysis. The comparison and trend estimation of haplotypes were assessed by unpaired T-test and linear regression analysis respectively with GraphPad Prism 5.

## Results

### Molecular surveillance of *Pfdhfr* and *Pfdhps* mutations

In total, 303 isolates from 2011 to 2019 were included for the analysis of *Pfdhfr* and *Pfdhps* mutations. Table 1 summarizes the different SNPs observed in the samples collected over these years. For *Pfdhfr*, mutations at different loci, including N51**I**, C59**R**, S108**N,** and I164**L**, were investigated. Of the 300 samples successfully genotyped for the *Pfdhfr* gene, 90.7% (272/300) harboured the mutant allele N51**I**, while six samples (2.0%) had a mixed type. For the *Pfdhfr* codon at 59, 84.7% (254/300) samples had the mutant allele C59**R**, and 9 (3%) had mixed genotypes. At codon 108, 94.7% (284/300) harboured a mutation, 3.0% (9/300) were wild type, and 2.3% (7/300) mixed infection were found. Only 1 sample out of 300 (0.3%) derived from Myanmar in 2011 had the highly drug resistant mutation I164**L**. However, we did not observe any mutation at codon 50 of *Pfdhfr*. For *Pfdhps*, mutations at different loci, including S436**A**, A437**G**, K540**E**, A581**G,** and A613**S**, were surveyed. Of the 290 (290/303, 95.7%) successful genotyped samples, 27.2% of the isolates harboured S436**A**, 79.0% harboured A437**G**, 21.7% harboured K540**E**, 9.0% harboured A581**G** and 11.0% harboured A613**S**. For wild type, 66.0% of the isolates harboured S436**A**, 13.1% A437**G**, 70.3% K540**E**, 85.5% A581**G** and 89.0% A613**S**, respectively. For mixed genotypes, 6.9% of the isolates harboured S436**A**, 7.9% harboured A437**G**, 7.9% harboured K540**E**, 5.5% harboured A581**G** and 0.3% harboured A613**S**.

**Table 1:** Observed the overall frequency of mutations in *Pfdhfr* and *Pfdhps*

Gene	Mutations	Wild type (%)	Mutation (%)	Mixed type (%)	Total
*Pfdhfr*	N51**I**	22 (7.3)	272 (90.7)	6 (2.0)	300
	C59**R**	37 (12.3)	254 (84.7)	9 (3.0)	300
	S108**N**	9 (3.0)	284 (94.7)	7 (2.3)	300
	I164**L**	299 (99.7)	1 (0.3)	0 (0.0)	300
*Pfdhps*	S436**A**	191 (65.9)	79 (27.2)	20 (6.9)	290
	A437**G**	38 (13.1)	229 (79.0)	23 (7.9)	290
	K540**E**	204 (70.3)	63 (21.7)	23 (7.9)	290
	A581**G**	248 (85.5)	26 (9.0)	16 (5.5)	290
	A613**S**	258 (89.0)	31 (11.0)	1 (0.3)	290

Mutations are shown in underline and bold

### Prevalence of the *Pfdhfr* and *Pfdhps* haplotypes

**Fig. 1:**
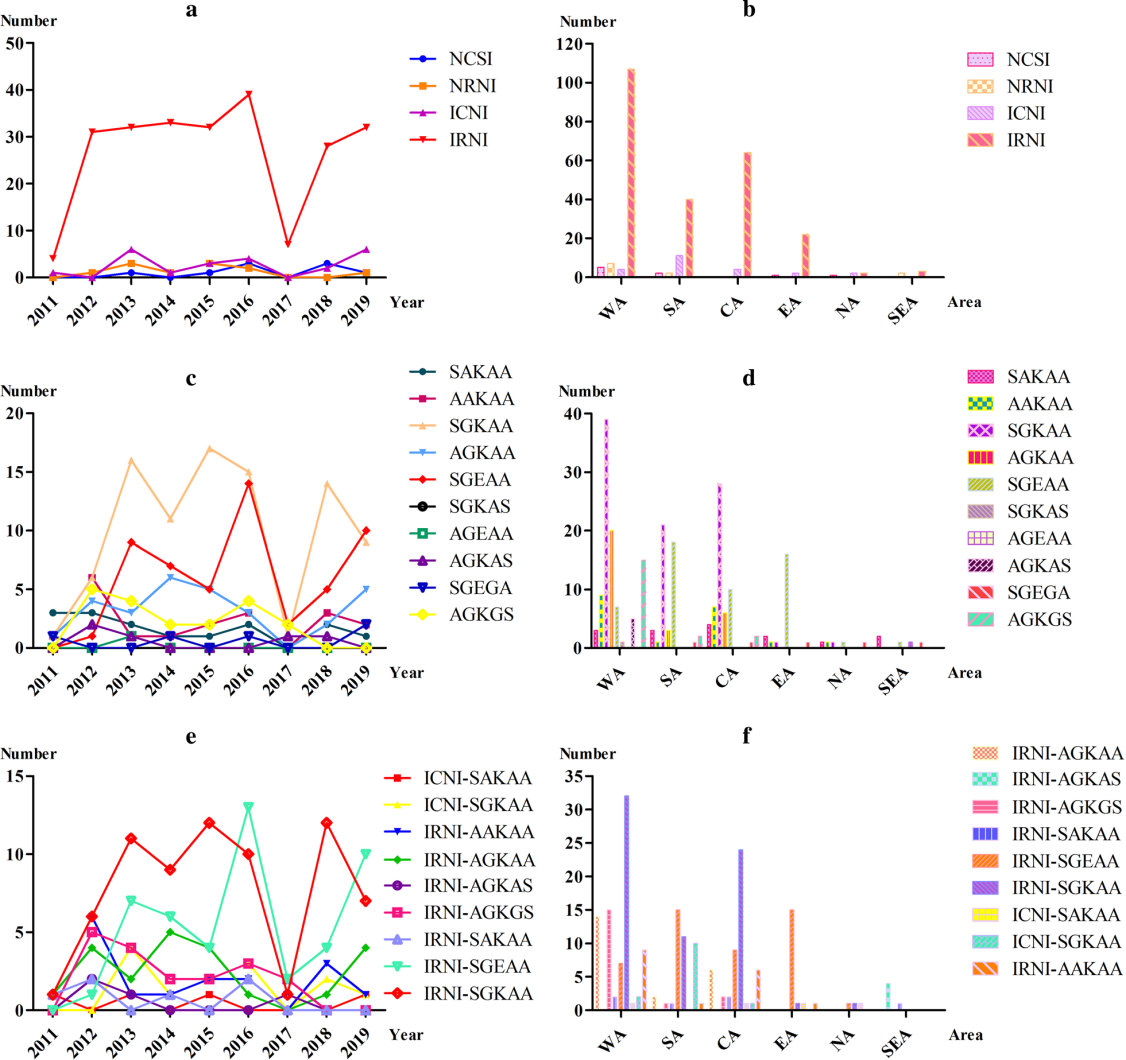
Observed haplotypes of *Pfdhfr* and *Pfdhps* in different years and areas. **a** The haplotypes of *Pfdhfr* in different years. **b** The haplotypes of *Pfdhfr* in different regions. **c** The haplotypes of *Pfdhps* in different years. **d** The haplotypes of *Pfdhps* in different areas. **e** The combined haplotypes of *Pfdhfr*-*Pfdhps* in different years. **f** The combined haplotypes of *Pfdhfr*-*Pfdhps* in different regions. The WA, SA, CA, EA, NA, and SEA represent West Africa, South Africa, Central Africa, East Africa, North Africa, and Southeast Asia, respectively

In total, five unique haplotypes at the *Pfdhfr* locus and 11 haplotypes at the *Pfdhps* locus were found. The prevalence of the *Pfdhfr* and *Pfdhps* haplotypes in different years and various geographical regions are illustrated in Fig. 1. The *Pfdhfr* mutations were the most prevalent, with the triple mutation (**IRN**I) in almost 84.4% (238/282). Compared to the haplotypes of NCSI, **I**C**N**I, and N**RN**I, the **IRN**I displayed a higher prevalence during 2011–2019 (*P* < 0.0001, *P* < 0.0001, and *P* < 0.0001, respectively) (Fig. 1a). As the predominant haplotype, the **IRN**I was found in all geographical areas in Africa and SEA (Fig. 1b). For NCSI (wild type), the frequency has increased from none in 2011 and 2012 to 9.1% (3/33) in 2018, and then reduced to 2.5% (1/40) in 2019. The relative frequencies of the **I**C**N**I mutations decreased from 16.67% (1/6) in 2011 to 0 in 2012 and increased to 14.3% (6/42) in 2013; finally stabilized at 15.0% (6/40) in 2019. For N**RN**I, there was only a marginal difference over these years (F = 0.6316, P = 0.4529). The allele with quadruple mutations (**IRNL**), which conferred a high level of resistance to antifolates, was only found at a low frequency (0.3%, 1/300) in 2011. For *Pfdhps*, the predominant haplotype was single-mutant S**G**KAA (37.8%, 90/238). It was increased from 14.3% (1/7) in 2011 to 30.0% (9/30) in 2019 (Fig. 1c). Although a decreasing trend was observed in the prevalence of the quadruple-mutant **AG**K**GS** from 18.5% in 2012 to 9.8% in 2016 and finally to 0% in 2019, these differences were not statistically significant (F = 0.0063, *P* = 0.9391). Similarly, the prevalence of the *Pfdhps* wild type SAKAA genotype has decreased from 42.9% in 2011 to 3.33% in 2019, but it was not statistically significant (F = 4.456, *P* = 0.0727). The *Pfdhps* double-mutant **AG**KAA genotype decreased from 14.3% in 2011 to 7.3% in 2016 and finally increased to 16.7% in 2019 (F = 0.6879, *P* = 0.4342). A concomitant increase in the prevalence of the *Pfdhps* double-mutant S**GE**AA genotype was observed, increasing in prevalence from 3.7% in 2012 to 33.3% in 2019, which was statistically significant (F = 9.034, *P* = 0.0198) (Fig. 1c). As the predominant haplotype, the S**G**KAA was mostly found in WA, CA, and SA with a prevalence of 93.4, 47.5, and 42.9%, respectively (Fig. 1d). S**GE**AA, was mostly distributed in SA (36.7%), EA (76.2%), and CA (17.0%) (Fig. 1d).

### Prevalence of *Pfdhfr* and *Pfdhps* combination haplotypes

A total of 230 samples were subjected to combined haplotypes analysis. The 28 haplotypes were verified by combining both genes. The most common haplotype was **IRN**I-S**G**KAA with a 30.0% (69/230) frequency, followed by **IRN**I-S**GE**AA (20.4%, 47/230), **IRN**I-**AG**KAA (9.8%, 22/230), and **IRN**I-**AG**K**GS** (7.8%, 18/230). Moreover, the frequency distribution of the different *Pfdhfr-Pfdhps* haplotypes was compared among the analysed samples collected at different times. The results showed that the most prevalent haplotype observed during the study period was **IRN**I-S**G**KAA, which also remained the most frequent one in WA (32.7%), SA (23.4%), and CA (44.4%) (Fig. 1e). For **IRN**I-S**GE**AA, an increasing trend was detected during 2011–2016, and 2017–2019, respectively (Fig. 1e). For regional distribution, **IRN**I-S**G**KAA was mainly distributed in WA (32.7%), CA (44.4%), and SA (23.4%) (Fig. 1F). Similarly, **IRN**I-S**GE**AA was mainly found in EA (71.4%) and SA (31.9%), followed by CA (16.7%) and WA (7.1%) (Fig. 1f). An additional 20 minor haplotypes with a prevalence of less than 2% constituted only 9.1% (21/227) of the overall haplotypes (Additional file 1: Table S1 and Additional file 2: Table S2).

## Discussion

Globally, strategies for malaria control have substantially reduced the disease burden in the last few decades. Soon afterward, several nations in Asia (particularly China), Africa, and Latin America began advancing towards malaria elimination [21–23]. However, imported malaria from Africa and SEA has affected and delayed the progress of malaria elimination in China. Furthermore, drug-resistant *P. falciparum p*arasites will become a significant challeng influencing the process of malaria control, elimination, and eradication. The SNPs in the *Pfdhfr* and *Pfdhps* genes are linked to the failure of SP treatment against uncomplicated *P. falciparum* malaria and have been documented in Africa and SEA for several decades [10, 11]. However, there are no such data to support drug policies in nonendemic areas with imported malaria in China particularly Wuhan [24]. To determine whether parasites carrying these polymorphisms exist in Wuhan, molecular surveillance were conducted targeting *Pfdhfr* and *Pfdhps* gene polymorphisms in imported clinical isolates.

For *Pfdhfr*, the critical event in the development of pyrimethamine resistance is a mutation in codon 108 that changes serine (S) to asparagine (N), resulting in partial pyrimethamine resistance. Further mutations at N51**I** and/or C59**R** increase the level of pyrimethamine resistance [25]. Under continuous pyrimethamine selective drug pressure, the SNP adaptations in our data have also followed this rule. The current survey demonstrated an extremely high prevalence (> 84%) of three mutations (N51**I**, C59**R,** and S108**N**) in *P. falciparum* clinical isolates imported from Africa and SEA. In Africa, *Pfdhfr* nonsynonymous polymorphisms have also been reported at high frequencies in isolates from Uganda [26], Angola [27], the Democratic Republic of the Congo [12], Nigeria [28], and Sierra Leone [29]. Parasitic infections carrying the *Pfdhfr* triple mutant (**IRN**I) are significantly more likely to be resistant to SP treatment than infections with fewer *Pfdhfr* mutations [30]. Most of the isolates in our dataset had the triple mutant allele **IRN**I (84.4%, 238/282), indicating that pyrimethamine resistance remains at a relatively high level in Africa. However, no mutations in codons 50 and 164 of *Pfdhfr* were detected in samples collected on the African continent. An additional mutation, I164**L**, confers an elevated level of pyrimethamine resistance that could render SP invalid [27]. Although the *Pfdhfr* I164**L** mutation was first reported from Kenya [31] and then was found in Madagascar [32] and the Central African Republic [33], it was not detected in Africa in the present study. Furthermore, only one sample (0.3%, 1/300) with the I164**L** mutation was found in 2011 from SEA (Myanmar).

For *Pfdhps*, as the key mutation associated with sulfadoxine resistance, a single amino acid residue changes from alanine (A) to glycine (G) at codon 437 of *Pfdhps* [34]. The A437**G** selection by SP has been previously described during IPTi [35]. As the most frequent mutation of *Pfdhps*, our findings illustrate the high prevalence of A437**G** at 79.0% (229/290), which has also been reported to be nearly at a saturation level in most African countries [27]. Furthermore, a higher proportion of A437**G** has been detected at 75.6% in Gabon [36], 87.9% in Kenya [37], 97.6% (1416/1451) in Congo [12], and 96.4% (27/28) in Nigeria [28]. Thus, it needs to be kept in mind that a high prevalence of SP-resistant parasites is present in these regions. More attention should be given to SP drug resistance surveillance, both in these countries and in nonedemic areas, particularly Wuhan, which is influenced by imported malaria from endemic areas. Compared to the high prevalence of *Pfdhfr* mutations, a low prevalence (< 30%) of four mutant alleles (S436**A**, K540**E**, A581**G**, and A613**S**) in *Pfdhps* was detected. It has been reported that *Pfdhps* K540**E** has a low prevalence in Central and West Africa [9, 14, 28, 33]. In contrast, the K540**E** mutation is common in East Africa [38, 39], similar to our findings. The *Pfdhps* A581**G** and A613**S/T** mutations have been detected at a low prevalence in WA and EA, but a rapid emergence of these mutations has been described in Kenya and Uganda [25, 33, 40]. Apart from Nigeria and Cameroon, these mutations have not been found in CA [41]. In Cameroon, there is an increasing trend in the prevalence of the *Pfdhps* A581**G** and A613**S** mutations [33]. In the current study, the prevalence of A581**G** and A613**S** were generally consistent with these observations in Africa.

Parasites carrying all five mutations, the *Pfdhfr* triple mutant (N51**I** + C59**R** + S108**N**) and the *Pfdhps* double mutant (A437**G** and K540**E**), commonly called the quintuple mutation (**IRN**I-S**GE**AA), have been strongly associated with SP treatment failure in sub-Saharan Africa [42–45]. Alarmingly, the present study found 18.54% of tested isolates harboured fully resistant (**IRN**I-S**GE**AA), which is common in EA [34, 46]. Additional mutations in *Pfdhfr* I164**L** and *Pfdhps* A581**G** have been associated with a high level of SP resistance and failure [47]. However, a quintuple mutant named “super-resistant genotypes” is linked with a more than triple enhancement of therapeutic failure [47]. The **IRN**I-S**GEG**A forming the sextuple haplotype has been connected with an optimal resistance effect, referred to as the “super-resistant genotype” [37]. Three isolates of such genotype are found in our data, which is of concern. In addition, it is noteworthy that the other combined haplotypes, including the quintuple mutant (**IRN**I-**AG**KAA, **IRN**I-S**G**KA**S**, **I**C**N**I-S**GEG**A), the sextuple mutant (**IRN**I**-AG**KA**S**, N**RN**I-**AG**K**GS**, **IRN**I-**AGE**AA), and the septuple mutant (**IRN**I-**AG**K**GS**, **IRNL**-S**GEG**A) were also detected in the current data. Previous studies revealed that **IRN**I-S**GEG**A, **IRN**I-**AGE**AA has been highly associated with a lack of IPTp-SP efficacy [48]. It was illustrated that such genotypes were widely distributed in Tanzania, in line with our study, where one isolate harboured the sextuple haplotype (**IRN**I-S**GEG**A), which happened to come from Tanzania [48]. Interestingly, the septuple mutant haplotype (**IRN**I-**AG**K**GS**) accounts for a certain proportion of our findings, which is similar to a previous study reported in Nigeria [3]. This demonstrates that SP resistance remains at a moderate level in Africa. Although SP is recommended as an effective anti-malarial drug used for the vulnerable population [35], more attention needs to be paid to these mutations profiles.

## Conclusions

In conclusion, the present study reports the persistence of *P. falciparum* parasites with *Pfdhfr* and *Pfdhps* mutations associated with SP resistance in migrant workers returning from Africa and SEA to Wuhan, central China. These findings provide fundamental prevalence data that enable a policy-making organization to directly determine the best measures and strategies for malaria control and elimination.

## Supplementary information

**Additional file 1: Table S1.** Haplotypes distribution of *Pfdhfr* and *Pfdhps* in a different country during 2011-2019.

**Additional file 2: Table S2.** The combined haplotypes distribution of *Pfdhfr* and *Pfdhps* in different years and areas.

## Data Availability

The datasets analysed in this study are available from the corresponding author on reasonable request.

## Abbreviations

A: Alanine
G: Glycine
N: Asparagine
S: Serine
ACTs: Artemisinin-based combination therapies
CQ: Chloroquine
gDNA: Genomic DNA
*Pfdhps*: *P. falciparum* Dihydropteroate synthase
*Pfdhfr*: *P. falciparum* Dihydrofolate reductase
IPTi: Intermittent preventive treatment in infants
IPTp: Intermittent preventive treatment pregnant women
RDTs: Rapid diagnostic tests
Pf-HRP2: *P. falciparum* Specific Histidine-rich protein 2
pLDH: Plasmodium lactate dehydrogenase
SMC: Seasonal Malaria Chemoprevention
SEA: Southeast Asia
SP: Sulfadoxine-pyrimethamine
SNPs: Single-nucleotide polymorphisms
